# Tetra­kis(4-cyano­pyridine)­palladium(II) bis­(trifluoro­methane­sulfonate)

**DOI:** 10.1107/S1600536810027704

**Published:** 2010-07-17

**Authors:** Rafael A. Adrian, David M. Gonzalez, Edward R. T. Tiekink, Judith A. Walmsley

**Affiliations:** a Department of Chemistry, University of the Incarnate Word, San Antonio, TX 78209, USA; b Department of Chemistry, University of Malaya, 50603 Kuala Lumpur, Malaysia; c Department of Chemistry, University of Texas at San Antonio, San Antonio, TX 78249, USA

## Abstract

The title salt, [Pd(C_6_H_4_N_2_)_4_](CF_3_SO_3_)_2_, comprises Pd(4-cyano­pyridine)_4_ dications balanced by two trifluoro­methane­sulfonate anions. The Pd^II^ atom lies in a square-planar geometry defined by four N atoms which form equivalent Pd—N inter­actions. The 4-cyano­pyridine ligands are twisted out of the N_4_ plane, forming dihedral angles ranging from 66.5 (2) to 89.9 (2)°. In the crystal packing, columns of edge-to-edge dications define channels in which reside the anions. A range of C—H⋯N and C—H⋯O hydrogen-bonding interactions stabilizes the crystal packing.

## Related literature

For related palladium(II) complexes with 4-cyano­pyridine, see: Kopylovich *et al.* (2009 ▶); Lang *et al.* (2006 ▶); Taher *et al.* (2006 ▶).
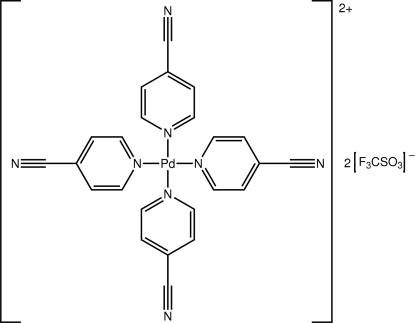


## Experimental

### 

#### Crystal data

[Pd(C_6_H_4_N_2_)_4_](CF_3_O_3_S)_2_
*M* *_r_* = 820.99
Monoclinic, 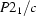

*a* = 18.550 (4) Å
*b* = 9.2993 (19) Å
*c* = 20.688 (4) Å
β = 114.55 (3)°
*V* = 3246.1 (14) Å^3^
*Z* = 4
Mo *K*α radiation
μ = 0.79 mm^−1^
*T* = 153 K
0.20 × 0.20 × 0.20 mm

#### Data collection

Rigaku AFC12/SATURN724 diffractometer
Absorption correction: multi-scan (*ABSCOR*; Higashi, 1995 ▶) *T*_min_ = 0.829, *T*_max_ = 1.000
12799 measured reflections
5478 independent reflections
5000 reflections with *I* > 2σ(*I*)
*R*_int_ = 0.035

#### Refinement

*R*[*F* ^2^ > 2σ(*F* ^2^)] = 0.048
*wR*(*F* ^2^) = 0.128
*S* = 1.14
5478 reflections
442 parameters
H-atom parameters constrained
Δρ_max_ = 1.05 e Å^−3^
Δρ_min_ = −0.74 e Å^−3^

### 

Data collection: *CrystalClear* (Molecular Structure Corporation & Rigaku, 2005 ▶); cell refinement: *CrystalClear*; data reduction: *CrystalClear*; program(s) used to solve structure: *SHELXS97* (Sheldrick, 2008 ▶); program(s) used to refine structure: *SHELXL97* (Sheldrick, 2008 ▶); molecular graphics: *ORTEP-3* (Farrugia, 1997 ▶) and *DIAMOND* (Brandenburg, 2006 ▶); software used to prepare material for publication: *publCIF* (Westrip, 2010 ▶).

## Supplementary Material

Crystal structure: contains datablocks global, I. DOI: 10.1107/S1600536810027704/ez2223sup1.cif

Structure factors: contains datablocks I. DOI: 10.1107/S1600536810027704/ez2223Isup2.hkl

Additional supplementary materials:  crystallographic information; 3D view; checkCIF report

## Figures and Tables

**Table 1:** Selected bond lengths (Å)

Pd—N1	2.027 (4)
Pd—N3	2.031 (4)
Pd—N5	2.029 (4)
Pd—N7	2.027 (4)

**Table 2:** Hydrogen-bond geometry (Å, °)

*D*—H⋯*A*	*D*—H	H⋯*A*	*D*⋯*A*	*D*—H⋯*A*
C4—H4⋯N8^i^	0.95	2.56	3.489 (8)	166
C5—H5⋯O2	0.95	2.44	3.159 (6)	132
C7—H7⋯O5^ii^	0.95	2.52	3.202 (6)	129
C8—H8⋯N6^iii^	0.95	2.53	3.441 (8)	160
C13—H13⋯O5^ii^	0.95	2.33	3.134 (7)	141
C16—H16⋯N6^iv^	0.95	2.61	3.403 (8)	142
C22—H22⋯O3^v^	0.95	2.52	3.170 (7)	126
C23—H23⋯O2	0.95	2.34	3.163 (7)	145

Symmetry codes: (i) 
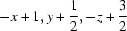
; (ii) 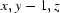; (iii) 
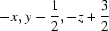
; (iv) 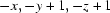; (v) 
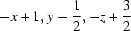
.

**Fig. 1.**
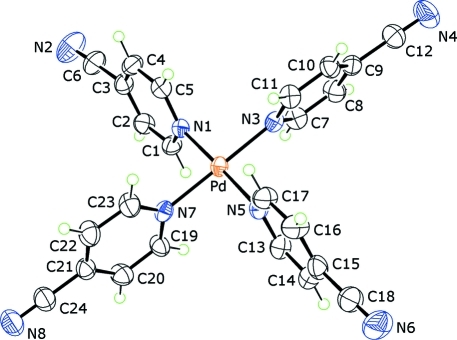
Molecular structure of the cation in (I) showing atom-labelling scheme and displacement ellipsoids at the 50% probability level.

**Fig. 2.**
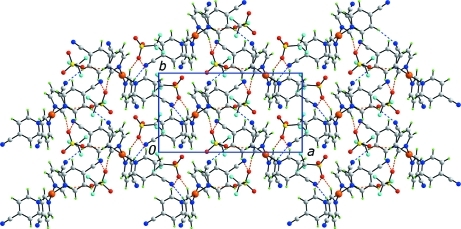
Supramolecular 2-D array (I) mediated by C–H···N and C–H···O contacts (blue and orange dashed lines, respectively). Colour code: Pd, orange; S, yellow; O, red; N, blue; C, grey; H, green.

**Fig. 3.**
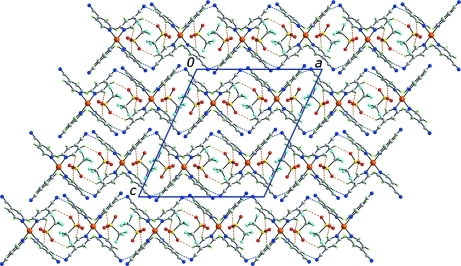
Stacking of layers in (I), highlighting the formation of channels by the dications in which reside the anions. The C–H···N and C–H···O contacts are shown as blue and orange dashed lines, respectively. Colour code: Pd, orange; S, yellow; O, red; N, blue; C, grey; H, green.

## supplementary crystallographic information

### Comment

While studying the palladium-catalyzed hydration of nitriles, we sought to crystallize palladium(II) complexes with 4-cyanopyridine, similar to those created by Kopylovich *et al.* (2009), Lang *et al.* (2006) and Taher *et al.* (2006). The resulting yellow crystals that formed, (I), were found not to contain any ethylenediamine, but contained a palladium(II) center where the inner coordination sphere is occupied by 4-cyanopyridine ligands.

The molecular structure of (I) comprises a Pd(4-cyanopyridine)_4_ dication, Fig. 1, and two trifluoromethanesulfonate anions. The palladium atom lies in a square planar geometry defined by four pyridine-N atoms which form experimentally equivalent Pd–N bond distances, Table 1. The palladium atom lies in the least-squares plane through the N_4_ donor set with the r.m.s. deviation for the PdN_4_ atoms being 0.021 Å. For steric reasons, each of the 4-cyanopyridine molecules is twisted with respect to the N_4_ plane, forming dihedral angles with it of 72.21 (19), 66.5 (2), 80.1 (2), and 89.9 (2) ° for the N1-, N3-, N5-, and N7-pyridine rings, respectively.

In the crystal packing, molecules self-assemble into layers in the *ab* plane *via C*–*H*···N_cyano_ and C–H···O interactions, Table 2. The resulting 2-D array, Fig. 2, can be described as comprising rows of edge-to-edge complex dications that define channels in which reside the anions, Fig. 3.

### Experimental

Pd(ethylenediamine)(trifluoromethanesulfonate)_2_ was prepared by adding solid Ag(trifluoromethanesulfonate) to an aqueous solution of Pd(ethylenediamine)Cl_2_ (0.050 g, 0.21 mmol). After stirring for 1 h, the mixture was filtered to remove AgCl. 4-Cyanopyridine (0.090 g, 0.85 mmol) was added to the Pd(ethylenediamine)(trifluoromethanesulfonate)_2_ solution and heated at 323 K for 2 h. The solution was then allowed to evaporate at room temperature, yielding a yellow solid. X-ray diffraction quality crystals were obtained by vapor diffusion of diethyl ether over a CH_3_CN solution of the title complex, (I). (0.026 g, 15% yield). IR (cm^-1^, solid): ν(═CH) 3112 (w), 3083(w), 3022(w); ν(CN) 2244 (w); νs(CF_3_) 1220 (*s*), νs(SO_3_) 1028 (*s*). *M*.pt.: 498 K (dec.) with melting at 523 K.

### Refinement

The H-atoms were included in the refinement in the riding model approximation (C–H = 0.95 Å) with *U*_iso_(H) set to 1.2*U*_eq_(carrier atom). The maximum and minimum residual electron density peaks of 1.05 and 0.74 e Å^-3^, respectively, were located 0.71 Å and 0.63 Å from the C11 and S1 atoms, respectively.

### Crystal data

**Table:**

[Pd(C_6_H_4_N_2_)_4_](CF_3_O_3_S)_2_	*F*(000) = 1632
*M**_r_* = 820.99	*D*_x_ = 1.680 Mg m^−^^3^
Monoclinic, *P*2_1_/*c*	Mo *K*α radiation, λ = 0.71073 Å
Hall symbol: -P 2ybc	Cell parameters from 4545 reflections
*a* = 18.550 (4) Å	θ = 2.4–26.7°
*b* = 9.2993 (19) Å	µ = 0.79 mm^−^^1^
*c* = 20.688 (4) Å	*T* = 153 K
β = 114.55 (3)°	Block, pale-yellow
*V* = 3246.1 (14) Å^3^	0.20 × 0.20 × 0.20 mm
*Z* = 4	

### Data collection

**Table:**

Rigaku AFC12K/SATURN724 diffractometer	5478 independent reflections
Radiation source: fine-focus sealed tube	5000 reflections with *I* > 2σ(*I*)
graphite	*R*_int_ = 0.035
ω scans	θ_max_ = 25.0°, θ_min_ = 2.4°
Absorption correction: multi-scan (*ABSCOR*; Higashi, 1995)	*h* = −22→20
*T*_min_ = 0.829, *T*_max_ = 1.000	*k* = −9→11
12799 measured reflections	*l* = −24→22

### Refinement

**Table:**

Refinement on *F*^2^	Primary atom site location: structure-invariant direct methods
Least-squares matrix: full	Secondary atom site location: difference Fourier map
*R*[*F*^2^ > 2σ(*F*^2^)] = 0.048	Hydrogen site location: inferred from neighbouring sites
*wR*(*F*^2^) = 0.128	H-atom parameters constrained
*S* = 1.14	*w* = 1/[σ^2^(*F*_o_^2^) + (0.0534*P*)^2^ + 6.3187*P*] where *P* = (*F*_o_^2^ + 2*F*_c_^2^)/3
5478 reflections	(Δ/σ)_max_ = 0.001
442 parameters	Δρ_max_ = 1.05 e Å^−^^3^
0 restraints	Δρ_min_ = −0.74 e Å^−^^3^

### Special details

**Table:**

Geometry. All s.u.'s (except the s.u. in the dihedral angle between two l.s. planes) are estimated using the full covariance matrix. The cell s.u.'s are taken into account individually in the estimation of s.u.'s in distances, angles and torsion angles; correlations between s.u.'s in cell parameters are only used when they are defined by crystal symmetry. An approximate (isotropic) treatment of cell s.u.'s is used for estimating s.u.'s involving l.s. planes.
Refinement. Refinement of *F*^2^ against ALL reflections. The weighted *R*-factor *wR* and goodness of fit *S* are based on *F*^2^, conventional *R*-factors *R* are based on *F*, with *F* set to zero for negative *F*^2^. The threshold expression of *F*^2^ > 2σ(*F*^2^) is used only for calculating *R*-factors(gt) *etc*. and is not relevant to the choice of reflections for refinement. *R*-factors based on *F*^2^ are statistically about twice as large as those based on *F*, and *R*- factors based on ALL data will be even larger.

### Fractional atomic coordinates and isotropic or equivalent isotropic displacement parameters (Å^2^)

**Table:**

	*x*	*y*	*z*	*U*_iso_*/*U*_eq_	
Pd	0.254695 (18)	0.02757 (4)	0.766848 (17)	0.02851 (14)	
S1	0.39867 (7)	0.39076 (14)	0.77597 (7)	0.0421 (3)	
S2	0.11861 (7)	0.66442 (14)	0.77812 (7)	0.0384 (3)	
F1	0.3763 (3)	0.3677 (5)	0.6440 (2)	0.0951 (14)	
F2	0.4595 (3)	0.5294 (4)	0.7007 (3)	0.0944 (14)	
F3	0.4938 (3)	0.3095 (5)	0.7191 (2)	0.0874 (13)	
F4	−0.03138 (19)	0.7372 (4)	0.7237 (2)	0.0733 (10)	
F5	−0.0103 (2)	0.5195 (4)	0.7061 (3)	0.0954 (15)	
F6	0.0144 (2)	0.6830 (5)	0.64715 (19)	0.0856 (12)	
O1	0.3355 (2)	0.4938 (4)	0.7542 (2)	0.0559 (10)	
O2	0.3714 (2)	0.2450 (4)	0.7711 (2)	0.0571 (10)	
O3	0.4674 (2)	0.4248 (5)	0.8390 (2)	0.0701 (13)	
O4	0.1119 (2)	0.6364 (5)	0.8435 (2)	0.0661 (12)	
O5	0.1394 (2)	0.8100 (4)	0.7697 (2)	0.0499 (9)	
O6	0.1616 (2)	0.5589 (4)	0.7574 (2)	0.0545 (10)	
N1	0.3470 (2)	−0.0809 (4)	0.84066 (18)	0.0300 (8)	
N2	0.5954 (3)	−0.3914 (7)	1.0098 (3)	0.0771 (17)	
N3	0.2329 (2)	0.1341 (4)	0.84253 (19)	0.0306 (8)	
N4	0.1540 (3)	0.4235 (6)	1.0271 (3)	0.0672 (14)	
N5	0.1614 (2)	0.1304 (4)	0.69095 (19)	0.0316 (8)	
N6	−0.0892 (3)	0.4022 (6)	0.4987 (3)	0.0653 (14)	
N7	0.2771 (2)	−0.0774 (4)	0.69126 (19)	0.0318 (8)	
N8	0.3503 (3)	−0.3732 (6)	0.5062 (2)	0.0570 (12)	
C1	0.3401 (3)	−0.2230 (5)	0.8488 (2)	0.0354 (10)	
H1	0.2905	−0.2682	0.8230	0.042*	
C2	0.4029 (3)	−0.3051 (5)	0.8936 (2)	0.0392 (11)	
H2	0.3966	−0.4052	0.8988	0.047*	
C3	0.4752 (3)	−0.2391 (6)	0.9307 (2)	0.0380 (11)	
C4	0.4820 (3)	−0.0920 (6)	0.9233 (2)	0.0405 (11)	
H4	0.5310	−0.0444	0.9490	0.049*	
C5	0.4169 (3)	−0.0165 (5)	0.8782 (2)	0.0349 (10)	
H5	0.4215	0.0843	0.8735	0.042*	
C6	0.5424 (3)	−0.3230 (6)	0.9757 (3)	0.0501 (13)	
C7	0.2062 (3)	0.0623 (6)	0.8847 (2)	0.0399 (11)	
H7	0.2007	−0.0392	0.8804	0.048*	
C8	0.1865 (3)	0.1317 (6)	0.9340 (3)	0.0437 (12)	
H8	0.1679	0.0793	0.9634	0.052*	
C9	0.1945 (3)	0.2796 (5)	0.9396 (2)	0.0360 (11)	
C10	0.2225 (3)	0.3537 (5)	0.8974 (2)	0.0374 (11)	
H10	0.2288	0.4552	0.9011	0.045*	
C11	0.2414 (3)	0.2758 (5)	0.8491 (2)	0.0364 (11)	
H11	0.2610	0.3258	0.8197	0.044*	
C12	0.1719 (3)	0.3602 (6)	0.9892 (3)	0.0493 (13)	
C13	0.0912 (3)	0.0632 (6)	0.6598 (3)	0.0390 (11)	
H13	0.0869	−0.0332	0.6731	0.047*	
C14	0.0258 (3)	0.1282 (6)	0.6098 (3)	0.0419 (12)	
H14	−0.0230	0.0779	0.5882	0.050*	
C15	0.0324 (3)	0.2699 (6)	0.5912 (2)	0.0424 (12)	
C16	0.1042 (3)	0.3394 (6)	0.6227 (3)	0.0460 (12)	
H16	0.1096	0.4360	0.6105	0.055*	
C17	0.1682 (3)	0.2669 (5)	0.6723 (2)	0.0406 (11)	
H17	0.2179	0.3142	0.6938	0.049*	
C18	−0.0355 (3)	0.3426 (6)	0.5394 (3)	0.0514 (14)	
C19	0.2442 (3)	−0.2063 (5)	0.6675 (2)	0.0383 (11)	
H19	0.2079	−0.2450	0.6845	0.046*	
C20	0.2615 (3)	−0.2836 (6)	0.6192 (3)	0.0425 (12)	
H20	0.2377	−0.3746	0.6028	0.051*	
C21	0.3145 (3)	−0.2260 (5)	0.5948 (2)	0.0370 (11)	
C22	0.3479 (3)	−0.0935 (6)	0.6189 (3)	0.0453 (12)	
H22	0.3839	−0.0519	0.6024	0.054*	
C23	0.3278 (3)	−0.0227 (6)	0.6675 (3)	0.0426 (12)	
H23	0.3509	0.0684	0.6847	0.051*	
C24	0.3344 (3)	−0.3078 (6)	0.5450 (3)	0.0462 (13)	
C25	0.4345 (4)	0.4002 (7)	0.7071 (4)	0.0596 (16)	
C26	0.0176 (3)	0.6496 (6)	0.7111 (3)	0.0548 (14)	

### Atomic displacement parameters (Å^2^)

**Table:**

	*U*^11^	*U*^22^	*U*^33^	*U*^12^	*U*^13^	*U*^23^
Pd	0.0277 (2)	0.0264 (2)	0.0324 (2)	0.00416 (13)	0.01340 (16)	0.00318 (13)
S1	0.0410 (7)	0.0374 (7)	0.0541 (7)	−0.0029 (6)	0.0259 (6)	−0.0035 (6)
S2	0.0339 (6)	0.0349 (7)	0.0495 (7)	0.0020 (5)	0.0202 (5)	0.0073 (5)
F1	0.120 (4)	0.117 (4)	0.054 (2)	−0.012 (3)	0.042 (2)	−0.002 (2)
F2	0.125 (4)	0.060 (3)	0.141 (4)	−0.020 (2)	0.098 (3)	0.008 (2)
F3	0.105 (3)	0.089 (3)	0.106 (3)	0.034 (3)	0.081 (3)	0.019 (2)
F4	0.0419 (18)	0.072 (3)	0.097 (3)	0.0145 (18)	0.0206 (18)	−0.004 (2)
F5	0.059 (2)	0.055 (2)	0.155 (4)	−0.0209 (19)	0.027 (3)	−0.007 (2)
F6	0.069 (2)	0.119 (4)	0.053 (2)	−0.011 (2)	0.0098 (18)	−0.002 (2)
O1	0.051 (2)	0.045 (2)	0.075 (3)	0.0109 (18)	0.029 (2)	−0.0001 (19)
O2	0.072 (3)	0.038 (2)	0.078 (3)	−0.0120 (19)	0.048 (2)	0.0012 (19)
O3	0.045 (2)	0.097 (4)	0.063 (3)	−0.002 (2)	0.017 (2)	−0.026 (3)
O4	0.060 (2)	0.092 (3)	0.060 (2)	0.019 (2)	0.038 (2)	0.026 (2)
O5	0.041 (2)	0.038 (2)	0.066 (2)	−0.0062 (16)	0.0177 (18)	0.0011 (17)
O6	0.048 (2)	0.048 (2)	0.074 (3)	0.0121 (18)	0.032 (2)	0.002 (2)
N1	0.0284 (19)	0.031 (2)	0.0310 (19)	0.0028 (16)	0.0124 (16)	−0.0009 (16)
N2	0.047 (3)	0.080 (4)	0.086 (4)	0.017 (3)	0.009 (3)	0.031 (3)
N3	0.0274 (18)	0.029 (2)	0.0354 (19)	0.0015 (16)	0.0130 (16)	0.0055 (16)
N4	0.080 (4)	0.079 (4)	0.057 (3)	0.001 (3)	0.042 (3)	−0.014 (3)
N5	0.032 (2)	0.029 (2)	0.0340 (19)	0.0043 (16)	0.0146 (16)	0.0028 (16)
N6	0.058 (3)	0.064 (3)	0.058 (3)	0.018 (3)	0.007 (3)	0.010 (3)
N7	0.0269 (19)	0.033 (2)	0.0353 (19)	0.0051 (16)	0.0123 (16)	0.0062 (17)
N8	0.063 (3)	0.065 (3)	0.049 (3)	0.010 (3)	0.030 (2)	−0.005 (2)
C1	0.032 (2)	0.031 (3)	0.041 (2)	−0.001 (2)	0.013 (2)	−0.002 (2)
C2	0.043 (3)	0.031 (3)	0.039 (3)	0.007 (2)	0.013 (2)	0.002 (2)
C3	0.035 (3)	0.045 (3)	0.032 (2)	0.011 (2)	0.012 (2)	0.005 (2)
C4	0.030 (2)	0.051 (3)	0.038 (3)	−0.003 (2)	0.011 (2)	0.000 (2)
C5	0.039 (3)	0.032 (3)	0.036 (2)	−0.001 (2)	0.018 (2)	0.002 (2)
C6	0.037 (3)	0.055 (4)	0.051 (3)	0.008 (3)	0.011 (2)	0.009 (3)
C7	0.043 (3)	0.038 (3)	0.040 (3)	−0.001 (2)	0.019 (2)	0.004 (2)
C8	0.049 (3)	0.048 (3)	0.040 (3)	−0.005 (2)	0.025 (2)	0.000 (2)
C9	0.032 (2)	0.046 (3)	0.029 (2)	0.004 (2)	0.0117 (19)	0.000 (2)
C10	0.040 (3)	0.033 (3)	0.038 (2)	0.006 (2)	0.016 (2)	−0.001 (2)
C11	0.041 (3)	0.032 (3)	0.041 (3)	0.001 (2)	0.021 (2)	0.006 (2)
C12	0.052 (3)	0.057 (4)	0.043 (3)	0.001 (3)	0.024 (3)	−0.003 (3)
C13	0.039 (3)	0.038 (3)	0.043 (3)	0.005 (2)	0.019 (2)	0.008 (2)
C14	0.035 (3)	0.048 (3)	0.043 (3)	0.003 (2)	0.016 (2)	0.005 (2)
C15	0.044 (3)	0.047 (3)	0.036 (2)	0.017 (2)	0.017 (2)	0.003 (2)
C16	0.053 (3)	0.031 (3)	0.044 (3)	0.005 (2)	0.009 (2)	0.006 (2)
C17	0.042 (3)	0.034 (3)	0.039 (3)	0.003 (2)	0.009 (2)	0.001 (2)
C18	0.051 (3)	0.049 (3)	0.046 (3)	0.015 (3)	0.011 (3)	0.002 (3)
C19	0.043 (3)	0.038 (3)	0.042 (3)	−0.007 (2)	0.026 (2)	−0.007 (2)
C20	0.048 (3)	0.036 (3)	0.046 (3)	−0.009 (2)	0.022 (2)	−0.007 (2)
C21	0.036 (2)	0.040 (3)	0.034 (2)	0.010 (2)	0.014 (2)	0.003 (2)
C22	0.048 (3)	0.044 (3)	0.055 (3)	−0.005 (2)	0.032 (3)	−0.002 (2)
C23	0.046 (3)	0.036 (3)	0.055 (3)	−0.007 (2)	0.030 (3)	−0.005 (2)
C24	0.049 (3)	0.052 (3)	0.041 (3)	0.008 (3)	0.022 (2)	0.001 (2)
C25	0.076 (4)	0.044 (4)	0.077 (4)	−0.003 (3)	0.050 (4)	0.002 (3)
C26	0.047 (3)	0.044 (3)	0.073 (4)	−0.007 (3)	0.024 (3)	−0.002 (3)

### Geometric parameters (Å, °)

**Table:**

Pd—N1	2.027 (4)	C2—H2	0.9500
Pd—N3	2.031 (4)	C3—C4	1.388 (7)
Pd—N5	2.029 (4)	C3—C6	1.437 (7)
Pd—N7	2.027 (4)	C4—C5	1.373 (7)
S1—O3	1.430 (4)	C4—H4	0.9500
S1—O1	1.434 (4)	C5—H5	0.9500
S1—O2	1.435 (4)	C7—C8	1.377 (7)
S1—C25	1.808 (6)	C7—H7	0.9500
S2—O4	1.433 (4)	C8—C9	1.383 (7)
S2—O5	1.438 (4)	C8—H8	0.9500
S2—O6	1.439 (4)	C9—C10	1.372 (7)
S2—C26	1.815 (6)	C9—C12	1.465 (7)
F1—C25	1.338 (8)	C10—C11	1.392 (6)
F2—C25	1.313 (7)	C10—H10	0.9500
F3—C25	1.325 (7)	C11—H11	0.9500
F4—C26	1.325 (7)	C13—C14	1.366 (7)
F5—C26	1.304 (7)	C13—H13	0.9500
F6—C26	1.335 (7)	C14—C15	1.393 (7)
N1—C5	1.344 (6)	C14—H14	0.9500
N1—C1	1.345 (6)	C15—C16	1.377 (7)
N2—C6	1.137 (7)	C15—C18	1.439 (7)
N3—C11	1.327 (6)	C16—C17	1.379 (7)
N3—C7	1.346 (6)	C16—H16	0.9500
N4—C12	1.135 (7)	C17—H17	0.9500
N5—C13	1.343 (6)	C19—C20	1.373 (7)
N5—C17	1.347 (6)	C19—H19	0.9500
N6—C18	1.146 (7)	C20—C21	1.385 (7)
N7—C23	1.330 (6)	C20—H20	0.9500
N7—C19	1.342 (6)	C21—C22	1.376 (7)
N8—C24	1.140 (6)	C21—C24	1.447 (7)
C1—C2	1.379 (6)	C22—C23	1.377 (7)
C1—H1	0.9500	C22—H22	0.9500
C2—C3	1.380 (7)	C23—H23	0.9500
			
N1—Pd—N7	87.80 (14)	C10—C9—C12	118.8 (5)
N1—Pd—N5	178.15 (15)	C8—C9—C12	121.1 (4)
N7—Pd—N5	90.70 (14)	C9—C10—C11	118.0 (5)
N1—Pd—N3	92.21 (14)	C9—C10—H10	121.0
N7—Pd—N3	179.53 (15)	C11—C10—H10	121.0
N5—Pd—N3	89.29 (14)	N3—C11—C10	122.4 (4)
O3—S1—O1	116.1 (3)	N3—C11—H11	118.8
O3—S1—O2	115.4 (3)	C10—C11—H11	118.8
O1—S1—O2	113.0 (2)	N4—C12—C9	179.4 (7)
O3—S1—C25	103.2 (3)	N5—C13—C14	122.4 (5)
O1—S1—C25	103.9 (3)	N5—C13—H13	118.8
O2—S1—C25	102.9 (3)	C14—C13—H13	118.8
O4—S2—O5	114.7 (3)	C13—C14—C15	118.5 (5)
O4—S2—O6	115.7 (3)	C13—C14—H14	120.8
O5—S2—O6	113.4 (2)	C15—C14—H14	120.8
O4—S2—C26	103.9 (3)	C16—C15—C14	119.4 (5)
O5—S2—C26	102.8 (2)	C16—C15—C18	120.5 (5)
O6—S2—C26	104.2 (3)	C14—C15—C18	120.1 (5)
C5—N1—C1	119.0 (4)	C15—C16—C17	119.2 (5)
C5—N1—Pd	121.6 (3)	C15—C16—H16	120.4
C1—N1—Pd	119.3 (3)	C17—C16—H16	120.4
C11—N3—C7	119.1 (4)	N5—C17—C16	121.3 (5)
C11—N3—Pd	120.7 (3)	N5—C17—H17	119.4
C7—N3—Pd	120.2 (3)	C16—C17—H17	119.4
C13—N5—C17	119.2 (4)	N6—C18—C15	179.0 (6)
C13—N5—Pd	119.8 (3)	N7—C19—C20	121.7 (4)
C17—N5—Pd	121.0 (3)	N7—C19—H19	119.1
C23—N7—C19	119.3 (4)	C20—C19—H19	119.1
C23—N7—Pd	120.2 (3)	C19—C20—C21	118.6 (5)
C19—N7—Pd	120.4 (3)	C19—C20—H20	120.7
N1—C1—C2	122.0 (4)	C21—C20—H20	120.7
N1—C1—H1	119.0	C22—C21—C20	119.7 (4)
C2—C1—H1	119.0	C22—C21—C24	121.3 (5)
C1—C2—C3	118.8 (5)	C20—C21—C24	119.1 (5)
C1—C2—H2	120.6	C21—C22—C23	118.3 (5)
C3—C2—H2	120.6	C21—C22—H22	120.8
C2—C3—C4	119.2 (4)	C23—C22—H22	120.8
C2—C3—C6	120.0 (5)	N7—C23—C22	122.3 (5)
C4—C3—C6	120.8 (5)	N7—C23—H23	118.8
C5—C4—C3	119.0 (5)	C22—C23—H23	118.8
C5—C4—H4	120.5	N8—C24—C21	179.4 (6)
C3—C4—H4	120.5	F2—C25—F3	107.5 (5)
N1—C5—C4	122.0 (5)	F2—C25—F1	106.5 (6)
N1—C5—H5	119.0	F3—C25—F1	108.1 (5)
C4—C5—H5	119.0	F2—C25—S1	112.5 (4)
N2—C6—C3	178.1 (7)	F3—C25—S1	111.7 (4)
N3—C7—C8	121.9 (5)	F1—C25—S1	110.2 (4)
N3—C7—H7	119.0	F5—C26—F4	107.6 (5)
C8—C7—H7	119.0	F5—C26—F6	106.6 (5)
C7—C8—C9	118.5 (5)	F4—C26—F6	107.5 (5)
C7—C8—H8	120.8	F5—C26—S2	112.4 (4)
C9—C8—H8	120.8	F4—C26—S2	112.2 (4)
C10—C9—C8	120.1 (4)	F6—C26—S2	110.3 (4)
			
N7—Pd—N1—C5	105.2 (3)	Pd—N5—C13—C14	178.3 (4)
N3—Pd—N1—C5	−74.3 (3)	N5—C13—C14—C15	−0.7 (7)
N7—Pd—N1—C1	−70.1 (3)	C13—C14—C15—C16	0.9 (7)
N3—Pd—N1—C1	110.4 (3)	C13—C14—C15—C18	−179.2 (5)
N1—Pd—N3—C11	115.5 (4)	C14—C15—C16—C17	−0.2 (7)
N5—Pd—N3—C11	−65.6 (4)	C18—C15—C16—C17	179.9 (5)
N1—Pd—N3—C7	−67.5 (3)	C13—N5—C17—C16	0.9 (7)
N5—Pd—N3—C7	111.5 (3)	Pd—N5—C17—C16	−177.5 (4)
N7—Pd—N5—C13	81.3 (3)	C15—C16—C17—N5	−0.7 (8)
N3—Pd—N5—C13	−99.2 (3)	C23—N7—C19—C20	0.1 (7)
N7—Pd—N5—C17	−100.3 (4)	Pd—N7—C19—C20	−176.4 (4)
N3—Pd—N5—C17	79.2 (4)	N7—C19—C20—C21	0.0 (7)
N1—Pd—N7—C23	−88.8 (4)	C19—C20—C21—C22	−0.4 (7)
N5—Pd—N7—C23	92.3 (4)	C19—C20—C21—C24	178.8 (5)
N1—Pd—N7—C19	87.7 (4)	C20—C21—C22—C23	0.7 (7)
N5—Pd—N7—C19	−91.3 (4)	C24—C21—C22—C23	−178.5 (5)
C5—N1—C1—C2	−1.2 (7)	C19—N7—C23—C22	0.2 (7)
Pd—N1—C1—C2	174.3 (3)	Pd—N7—C23—C22	176.7 (4)
N1—C1—C2—C3	−0.5 (7)	C21—C22—C23—N7	−0.6 (8)
C1—C2—C3—C4	1.6 (7)	O3—S1—C25—F2	−62.4 (6)
C1—C2—C3—C6	−177.7 (5)	O1—S1—C25—F2	59.2 (6)
C2—C3—C4—C5	−1.1 (7)	O2—S1—C25—F2	177.3 (5)
C6—C3—C4—C5	178.2 (4)	O3—S1—C25—F3	58.7 (5)
C1—N1—C5—C4	1.7 (7)	O1—S1—C25—F3	−179.7 (4)
Pd—N1—C5—C4	−173.6 (3)	O2—S1—C25—F3	−61.6 (5)
C3—C4—C5—N1	−0.6 (7)	O3—S1—C25—F1	178.8 (4)
C11—N3—C7—C8	0.7 (7)	O1—S1—C25—F1	−59.5 (5)
Pd—N3—C7—C8	−176.3 (4)	O2—S1—C25—F1	58.5 (5)
N3—C7—C8—C9	0.2 (7)	O4—S2—C26—F5	−65.3 (5)
C7—C8—C9—C10	−0.9 (7)	O5—S2—C26—F5	174.8 (5)
C7—C8—C9—C12	177.8 (5)	O6—S2—C26—F5	56.3 (5)
C8—C9—C10—C11	0.6 (7)	O4—S2—C26—F4	56.1 (5)
C12—C9—C10—C11	−178.0 (4)	O5—S2—C26—F4	−63.8 (5)
C7—N3—C11—C10	−1.0 (7)	O6—S2—C26—F4	177.7 (4)
Pd—N3—C11—C10	176.1 (3)	O4—S2—C26—F6	175.9 (4)
C9—C10—C11—N3	0.3 (7)	O5—S2—C26—F6	56.0 (5)
C17—N5—C13—C14	−0.2 (7)	O6—S2—C26—F6	−62.5 (5)

### Hydrogen-bond geometry (Å, °)

**Table:**

*D*—H···*A*	*D*—H	H···*A*	*D*···*A*	*D*—H···*A*
C4—H4···N8^i^	0.95	2.56	3.489 (8)	166
C5—H5···O2	0.95	2.44	3.159 (6)	132
C7—H7···O5^ii^	0.95	2.52	3.202 (6)	129
C8—H8···N6^iii^	0.95	2.53	3.441 (8)	160
C13—H13···O5^ii^	0.95	2.33	3.134 (7)	141
C16—H16···N6^iv^	0.95	2.61	3.403 (8)	142
C22—H22···O3^v^	0.95	2.52	3.170 (7)	126
C23—H23···O2	0.95	2.34	3.163 (7)	145

Symmetry codes: (i) −*x*+1, *y*+1/2, −*z*+3/2;  (ii) *x*, *y*−1, *z*; (iii) −*x*, *y*−1/2, −*z*+3/2; (iv) −*x*, −*y*+1, −*z*+1; (v) −*x*+1, *y*−1/2, −*z*+3/2.
